# Flavor profiling and gene expression studies of indigenous aromatic rice variety (*Mushk Budiji)* grown at different altitudes of Highland Himalayan regions

**DOI:** 10.1038/s41598-024-51467-z

**Published:** 2024-01-10

**Authors:** Ufaq Fayaz, Syed Zameer Hussain, Bazila Naseer, Syed Sheraz Mahdi, Javid Iqbal Mir, Alokesh Ghosh, Arun Jana, Nazrana Rafique Wani, Abida Jabeen, Fehim J. Wani, Sobiya Manzoor

**Affiliations:** 1 Division of Food Science and Technology, Sher-E-Kashmir University of Agriculture Sciences and Technology of Kashmir, Shalimar, 190025 India; 2 Division of Agronomy, Faculty of Agriculture, SKUAST-Kashmir, Wadura, J&K India; 3 Central Institute of Temperate Horticulture, Kashmir, Rangreth, J&K, 190005 India; 4 Centre for Development of Advanced Computing (C-DAC), Kolkata, 700001 India; 5 Division of Agricultural Economics & Statistics, Faculty of Agriculture, SKUAST-Kashmir, Wadura, J&K India

**Keywords:** Genetics, Chemistry

## Abstract

*Mushk Budiji*-an indigenous aromatic rice variety is usually grown at an altitude ranging from 5000 to 7000 ft above mean sea level in Highland Himalayas. This study was conducted to investigate the effects of altitude, soil nitrogen content and climatic conditions (temperature) of the selected locations on the flavor profile of *Mushk Budiji* using gas chromatography-mass spectroscopy (GC–MS) and electronic nose (E-nose). E-nose being rapid and non-destructive method was used to validate the results of volatile aromatic compounds obtained using GC–MS in *Mushk Budiji.* Around 35 aromatic compounds were identified in *Mushk Budiji* rice samples. Highest volatile peak area percentage (105.41%) was recorded for *Mushk Budji* grown at an altitude of 5216.53 ft. Highest E-nose score (2.52) was obtained at an altitude of 6299.21 ft. Over-expression of fatty acid degradation and linoleic acid metabolism genes was observed at higher altitudes, whereas lipid biosynthesis was negatively influenced by higher altitude. Fatty acid degradation and linoleic acid metabolism is responsible for the synthesis of volatile aromatic compounds in *Mushk Budiji*. This study will therefore be the path finder for investigating the intricate mechanism behind the role of altitude on aroma development in *Mushk Budiji* rice for future studies.

## Introduction

Rice (*Oryza sativa* L.) is the staple food for nearly half of the world’s population and is widely cultivated all over the world. Aroma is one of the most attractive characteristics of rice. The demand of aromatic rice is increasing globally^1^. In International markets, aromatic rice is considered to be the premium group of rice in the view of its enchanting grain quality, unique flavor and higher nutritional profile^2^. Aroma is an innate trait^3^ and its development is mainly affected by the genetic factors, growing conditions and post-harvest handling methods^4^. There are above 200 different aromatic compounds including 2-Acetyl-1-pyroline (2-AP), 2-acetyl-pyrrole, pyrolidone, pyridine etc. which are responsible for aroma of rice. 2-Acetyl-1-pyrroline (2AP) is considered as the major chemical compound responsible for the fragrance of aromatic rice^5^. The volatile aromatic compounds are synthesized in the aerial parts of rice seedling, in the early vegetative stage and final accumulation takes place in the seeds^6^. The main gene identified for regulating aroma in rice is fgr/badh2/Os2AP homologous to betaine aldehyde dehydrogenase (BADH) on chromosome number eight^7^. Proline is the main precursor of 2-AP, which is regulated by enzyme Δ1 -pyroline-5-carboxylic acid synthetase (P5CS). The 2AP synthesis in scented rice is attributed to the non-functionality of betaine aldehyde dehydrogenase (badh2) gene^8^. Accurate quantification of expression levels has been done for many genes through various throughput techniques. One such technique is the use of quantitative reverse transcription—polymerase chain reaction (qRTPCR or real-time RT-PCR) which allows weakly expressed genes to be accurately quantified^9^. Hinge et al.^10^ studied the expression analysis of major aroma volatile (2-AP)-related genes such as betaine aldehyde dehydrogenase 2 (badh2) and Δ1 -pyrolline-5-carboxylic acid synthetase (P5CS) using real-time PCR, in Basmati-370, Ambemohar-157 (non-basmati scented), and IR64 (non-scented) rice cultivars at vegetative and maturity stages. They reported maximum number of volatiles (72–58) at vegetative stage than at mature stage (54–39).

Several investigators have used E-nose and gas chromatography mass spectroscopy (GC–MS) techniques for determining the aroma profile of rice^11,12^. E-nose is a combination of gas sensors which mimics human nose. There occurs an irreversible change in the electrical properties such as conductivity, when the volatile aromatic compounds react with sensing material of gas sensor. These changes are then detected and characterized by pattern recognition algorithms to perform discrimination or classification of aromatic compounds^13^. Jana et al.^14^ successfully differentiated different aromatic rice samples using E-nose. Additionally, Zheng et al.^15^ also studied the rapid identification of four rice samples using an electronic nose (Cyranose-320) unit consisting of 32 polymer sensors. The Cyranose-320 was able to differentiate between varieties of rice, which was helpful for obtaining an accurate training model to improve identification capability. Gas chromatography mass spectrometry (GC–MS) is an instrumental technique, comprising a gas chromatograph (GC) coupled to a mass spectrometer (MS), by which complex mixtures of chemicals are separated, identified and quantified. Also, seven major active volatile compounds (hexanal, octanal, nonanal, (E)-2-octenal, decanal, 1-heptanol, and 1-octanol were detected in Jasmine rice by GC-MS^16^.

Basmati and Jasmine rice are the major aromatic rice groups. However, India is a treasure trove of scented rice beyond the basket of Basmati and Jasmine rice as well. Various scented rice varieties (*Ambemohar, Mullan Kazhama, Gobindo Bhog, Seerag Samba*, *Mushk Budiji, Radhuni Ragot and Chak Hao Amubijao*) are grown across the India^17^. Out of the different scented rice varieties, *Mushk Budiji*- the native aromatic rice variety of Highland Himalayan regions has prodigious demand in the international market due to its unique flavor and organoleptic appeal. *Mushk Budiji* is a short bold rice variety cultivated over an area of 10,000 ha, at an altitude above 5000 ft in Highland regions of Himalayas^18^. Genetic basis, variation in altitude, soil type and climatic conditions significantly effect the flavor profile of rice^19^. However, no studies have been conducted so far on *Mushk Budiji* rice in this direction. Furthermore, genetic mechanism of flavor development w.r.t altitude variation in case of *Mushk Budiji* rice is yet to be explored.

Therefore, in the present study flavor profiling of *Mushk Budiji* rice grown at eight different altitudes (ranging from 7053.80 to 5216.53 ft amsl) in Highland Himalayan regions was done using GC–MS and E-nose techniques. The novel transcriptomics approach was also used to understand the genetic basis of flavor development in *Mushk Budiji* rice, which identifies different pathways responsible for the production of volatile aromatic compounds contributing to the characteristic aroma in *Mushk Budiji.*

## Results and discussion

### Flavor profiling of rice

Flavor profile of *Mushk Budiji* rice grown at different altitudes is depicted in Table 1. Aroma volatiles usually include an oxygen-containing group, a nitrogen group, a sulfur group, and an aromatic group^20^. In this study, the identified species of volatile compounds (VOCs) in *Mushk Budiji* were mainly alcohols, aldehydes, hydrocarbons and other aromatic compounds. In general 35 volatile compounds were identified in *Mushk Budiji* rice, including 5 alcohols, 5 aldehydes, 1 ketone, 3 esters, 17 hydrocarbons and 4 other types. Among these VOC’s, aldehydes, and alcohols were more abundant in the aromatic rice samples grown at different altitudes. Aldehydes ranged from 6.33 to 29.09% and alcohols from 0.47 to 30.34% in rice samples grown at different locations (Table 1). Highest proportion of aldehydes and alcohols were recorded in L3 and L4 samples, respectively. Both these locations were characterized by different altitude and temperature (L3-6328.74 ft; T = 26.92 °C & L4-6299.21ft; T = 27.09 °C). Average rainfall received by L3 (67.94 mm) was also higher than the average rainfall received by L4 location (Table S1). In general aldehydes, alcohols and ketone based compounds were found higher in L4 samples as compared to other samples. It has been reported that aldehydes like hexanal & octanal produce fruity flavors while as nonanal produces citrus & fatty flavor in rice^21^. Fatty alcohols, such as 1-hexanol etc. are the secondary products of polyunsaturated fatty acids and produce a soft and sweet flavor in rice^22^. Among the fatty alcohols, flavor compounds like 1-hexanol (1.85%), propylene glycol (11.47%), 2-4-di-tert butylphenol (3.33%), silane diol dimethyl (13.69%) were recorded in aromatic rice grown at L4 (Table 1). Also hepatan-2-one, which is known to produce a characteristic fruity floral smell^23^ was found only in L4 (0.72%) sample. Acetoxyacetic acid-4- pentadecylester was found present only in L4 (1.49%) sample. However, 1,2-benzenedicarboxylic acid,bis (2-methylpropyl) ester was found highest (4.94%) in L7 followed by L6, L5, L8 and L1 sample while as 4-ethylbenzoicacid,cyclopentyl was found only in L3 (2.3%) and L8 (1.32%) samples. 2-pentyl furan which is known for its nutty and sweet aroma^8^ was found in all the samples except in L4 and L6 samples (Table 1).

**Table 1:** Relative contents of volatile compounds in *MushkBudiji* grown at different locations.

Compoud class	Compound name	Locations															
		L1		L2		L3		L4		L5		L6		L7		L8	
		RT (min)	Peak area (%)	RT	Peak area(%)	RT	Peak area (%)	RT	Peak area (%)	RT	Peak area(%)	RT	Peak area(%)	RT	Peak area(%)	RT	Peak area (%)
Aldehyde	Decanal	n.d	n.d	n.d	n.d	n.d	n.d	n.d	n.d	6.569	0.14	n.d	n.d	n.d	n.d	n.d	n.d
	Heptanal	3.341	0.43	n.d	n.d	n.d	n.d	n.d	n.d	3.341	0.29	n.d	n.d	3.342	0.77	n.d	n.d
	Octanal	4.431	0.45	n.d	n.d	n.d	n.d	2.356	11.9	4.432	0.35	n.d	n.d	4.433	1.27	n.d	n.d
	Nonanal	5.523	1.26	n.d	n.d	5.523	0.52	n.d	–	5.524	1.09	5.525	3.14	5.524	6.22	5.523	1.17
	Hexanal	2.356	18.76	2.353	9.92	2.356	5.51	2.352	17.19	2.356	7.13	2.356	12.27	2.356	6.12	2.355	6.54
Alcohols	1-hexanol	3.018	0.84	n.d	n.d	3.019	0.3	3.015	1.85	3.018	0.61			3.012	1.47	3.019	0.53
	Propylene glycol	n.d	n.d	n.d	n.d	n.d	n.d	1.996	11.47	n.d	n.d	n.d	n.d	n.d	n.d	n.d	n.d
	2,4-di-tert butylphenol	n.d	n.d	9.338	3.33	n.d	n.d	9.337	3.33	n.d	n.d	n.d	n.d	n.d	n.d	n.d	n.d
	3-pentanol,3-(1,1-dimethyl)-2,2,4,4-tetramethyl	n.d	n.d	n.d	n.d	6.342	0.17	n.d	n.d	6.342	0.12	-	-	6.344	0.94	n.d	n.d
	Silane diol dimethyl	n.d	n.d	1.67	11.89	n.d	n.d	1.671	13.69	n.d	n.d	2.109	6.74	2.3	3.76	n.d	n.d
Ketones	Heptan-2-one	n.d	n.d	n.d	n.d	n.d	n.d	3.214	0.72	n.d	n.d	n.d	n.d	n.d	n.d	n.d	n.d
Esters	Acetoxyacetic acid,4-pentadecylester	n.d	n.d	n.d	n.d	n.d	n.d	5.523	1.49	n.d	n.d	n.d	n.d	n.d	n.d	n.d	n.d
	1,2- benzenedicarboxylicacid,bis(2-methylpropyl)ester	12.231	0.38	n.d	n.d	n.d	n.d	n.d	n.d	12.23	1.35	12.33	3.7	12.231	4.94	12.23	0.43
	4-ethylbenzoicacid,cyclopentyl ester	n.d	n.d	n.d	n.d	12.231	2.3	n.d	n.d	n.d	n.d	n.d	n.d	n.d	n.d	3.558	1.32
Hydrocarbons	n-hexane	n.d	n.d	n.d	n.d	n.d	n.d	1.246	19.2	n.d	n.d	n.d	n.d	n.d	n.d	n.d	n.d
	cyclotrisiloxane,hexamethyl	n.d	n.d	n.d	n.d	n.d	n.d	n.d	n.d	n.d	n.d	n.d	n.d	5.166	1.3	6.89	0.60
	cyclopentasilicoxane,decamethyl	5.79	0.74	5.792	8.56	5.789	0.69	5.793	2.35	5.79	0.65	5.79	8.64	5.79	6.89	5.79	5.79
	cyclononasiloxane,octadecamethyl	n.d	n.d	n.d	n.d	n.d	n.d	n.d	n.d	n.d	n.d	11..428	0.76	n.d	n.d	n.d	n.d
	2,5-dimemethylhexane-2,5-dihydroperoxide	7.908	2.89	7.911	31.76	7.909	3.77	6.14	6.14	7.909	3.31	7.91	40.83	7.91	30.63	7.909	2.78
	cycloheptasilicoxane,tetradecamethyl	8.931	0.46	n.d	n.d	8.932	0.6	n.d	n.d	n.d	n.d	n.d	n.d	8.932	2.62	n.d	n.d
	1,5-heptadien-3-yne	n.d	n.d	n.d	n.d	n.d	n.d	n.d	n.d	n.d	n.d	n.d	n.d	n.d	n.d	2.1	83.56
	trisilicoxane,1,1,1,5,5,5-hexamethyl-3-[(trimethylsilyl)oxy]	n.d	n.d	4.154	1.2	n.d	n.d	n.d	n.d	n.d	n.d	n.d	n.d	n.d	n.d	n.d	n.d
	Toluene	2.103	80.92	n.d	n.d	2.102	83.65	n.d	n.d	2.102	82.56	n.d	n.d	2.101	3.14	-	-
	Naphthalene	6.444	0.62	n.d	n.d	n.d	n.d	n.d	n.d	6.445	0.19	n.d	n.d	6.446	1.5	6.444	0.49
	d-Limonene	n.d	n.d	n.d	n.d	n.d	n.d	4.794	2.04	n.d	n.d	n.d	n.d	n.d	n.d	n.d	n.d
	Dodecane	n.d	n.d	n.d	n.d	n.d	n.d	n.d	n.d	6.504	0.06	n.d	n.d	n.d	n.d	n.d	n.d
	Hexadecane	n.d	n.d	n.d	n.d	n.d	n.d	n.d	n.d	8.391	0.21	n.d	n.d	n.d	n.d	n.d	n.d
	Tetradecane	8.391	0.28	n.d	n.d	n.d	n.d	n.d	n.d	n.d	n.d	-	-	n.d	n.d	8.392	0.28
	cyclooctasiloxane, hexadecamethyl	10.255	0.17	n.d	n.d	10.256	0.38	n.d	n.d	n.d	n.d	10.257	1.53	10.256	0.19	10.256	0.19
	Cyclohexasilicoxanedodecamethyl	7.445	0.95	7.447	15.38	7.444	0.6	7.448	4.4	7.445	0.34	7.446	13.1	7.445	7.38	7.445	0.81
	Cyclotetrasiloxane,octamethyl	n.d	n.d	n.d	n.d	n.d	n.d	4.156	1.48	n.d	n.d	4.154	1.94	n.d	n.d	n.d	n.d
Others	2-pentyl furan	4.292	1.23	4.296	1.45	4.292	0.32	n.d	n.d	4.292	0.85	n.d	n.d	4.293	1.4	4.292	0.92
	2-Acetyl-1-pyroline	3.561	0.75	n.d	n.d	3.561	1	n.d	n.d	n.d	n.d	n.d	n.d	n.d	n.d	n.d	n.d
	Qunoline-1,2-dihydro-2,2,4-trimethyl	n.d	n.d	n.d	n.d	n.d	n.d	8.824	6.38	n.d	n.d	n.d	n.d	n.d	n.d	n.d	n.d
	Oxime methoxy phenyl	n.d	n.d	n.d	n.d	n.d	n.d	n.d	n.d	n.d	n.d	3.473	2.5	3.55	7.01	n.d	n.d

n.d not detected.

The highest number of hydrocarbons were identified in rice cultivated at L8. Also, aromatic rice grown at L8 exhibited higher concentration of total volatile compounds (total peak area percentage of 105.41%) in comparison to other samples. This area is located at an altitude of 5216.53 ft and received average rainfall of 41.10 mm with mean temperature of 29 °C. Prodhan et al.^2^ also reported that the aromatic rice genotypes contained more volatile compounds and displayed the maximum aroma score at temperatures surpassing their ambient conditions. These hydrocarbons are positively related to aroma traits and nutritional qualities^24^ and the accumulation of these volatile compounds in aromatic rice is closely related to the characteristics of its growth environment, such as climate, soil conditions, and altitude^25^, which is bound to vary with different cultivation areas^26^.

2-AP content was found to be present only in *Mushk Budiji* grown at L1 (0.75%) & L3 (1%) locations. Higher percentage of 2-AP in L3 samples is attributed to the low rainfall of 67.94 mm received by L3 in comparison to 74.29 mm of rainfall received by L1 location. This suggested that 2-AP levels were more influenced by rainfall conditions than by the altitude. Yoshihashi et al.^27^ also reported that 2AP levels in rice grains were highest when cultivated in regions with less water. Thus, it was observed that the amount of rainfall received by the samples affected the biosythesis, and accumulation of flavor volatiles in general and 2-AP in particular^28^. This could be due to the fact that rice leaves under water stress showed enhanced proline accumulation, which led to a considerable rise in 2-AP levels. Also, fragrant rice cultivars cultivated at different temperatures displayed varying degrees of 2AP accumulation^29^. Sansenya et al.^19^ also reported that 2-AP accumulation takes place in rain-fed areas at higher elevations accompanied by low air temperature.

Higher proportion of VOC’s were detected in aromatic rice grown at L8 (5216.53 ft) (Table 1), whereas the lowest number of volatiles were obtained from rice grown at L2 (6397.63 ft). This indicates that the aromatic rice samples harvested from locations at higher altitudes and receiving higher rainfall exhibited lower concentration of VOC’s than those from low altitudes with low rainfall. Rainfall is also an important factor in determining the yield and quality of aromatic rice. Furthermore, the mean temperatures and rainfall recorded for locations at higher altitudes was comparatively lower (26.1 °C; 69.21 mm) than the low altitude locations (29 °C; 41.10 mm), which indicated that high temperature and low rainfall was conducive to accumulate volatile aromatic compounds in *Mushk Budiji* rice. Thus, the accumulation of volatile compounds from aromatic rice samples harvested from different cultivation areas was potentially affected by different environmental conditions such as climate, soil conditions and altitude^25^. Indeed, previous authors have indicated that metabolite accumulation is substantially affected by environmental factors as well as by the genetic factors^30^.

Soil nitrogen data of L1 (7053.80) & L3 (6328.74 ft) locations showed nitrogen levels of 420 & 265 kg/ ha which suggested that 2-AP biosynthesis can occur both under low and high nitrogen soil conditions. However, it was reported that higher nitrogen content promotes 2-AP accumulation in rice^19^. Thus, this variation in our results can be attributed to different agro-climatic conditions and elevation levels tested.

### Gene expression of *Mushk Budiji*

**Figure 1:**
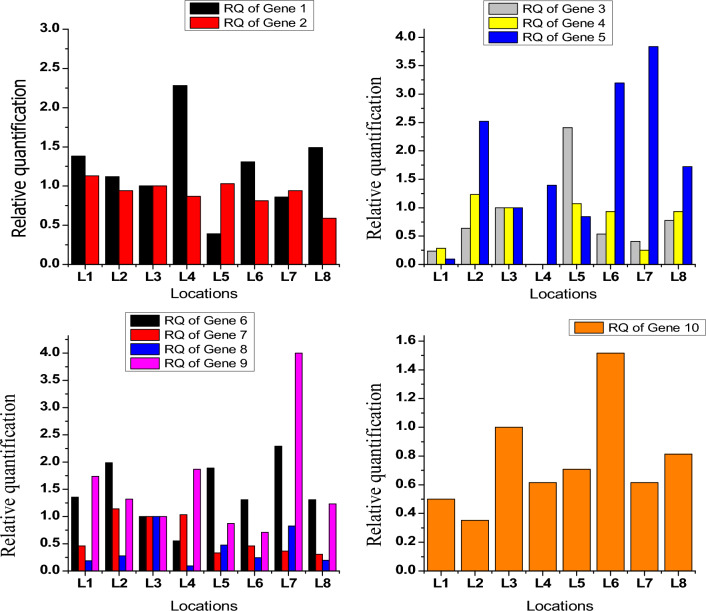
Relative quantification of fatty acid degradation, linoleic acid metabolism, ether lipid metabolism and badh2 genes in *Mushk Budiji* rice grown at different locations.

In the present study relative expression of candidate genes responsible for fatty acid degradation, linoleic acid metabolism and ether lipid metabolism leading to flavor development in *Mushk Budiji* rice at different elevations was studied (Fig. 1). The results elucidated the role of altitude in regulating their expression and finally leading to variation in aroma content. It was observed that at higher elevation genes responsible for fatty acid degradation (Gene- 1 & Gene- 2) and linoleic acid metabolism (Gene-3, Gene-4, Gene-5) were up-regulated. The results suggested that altitude significantly stimulated the expression of these genes (G-1, G-2, G-3, G-4, & G-5) which promoted the lipase activity, and thus produced free fatty acids and heterocyclic compounds. Over expression of genes at higher altitudes may be triggered by low temperature and higher light intensity^31,32^. Genes responsible for ether lipid metabolism (Gene-6, Gene-7, Gene-8 & Gene-9) and badh2 (Gene-10) were highly expressed in aromatic rice grown at lesser elevations (L2, L6, L7) revealing that the higher altitude inhibit the expression of these genes (G-6,G-7,G-8,G-9) which participate in the hydrolysis of lipases and degradation of phospholipases.

### Electronic nose analysis

**Figure 2:**
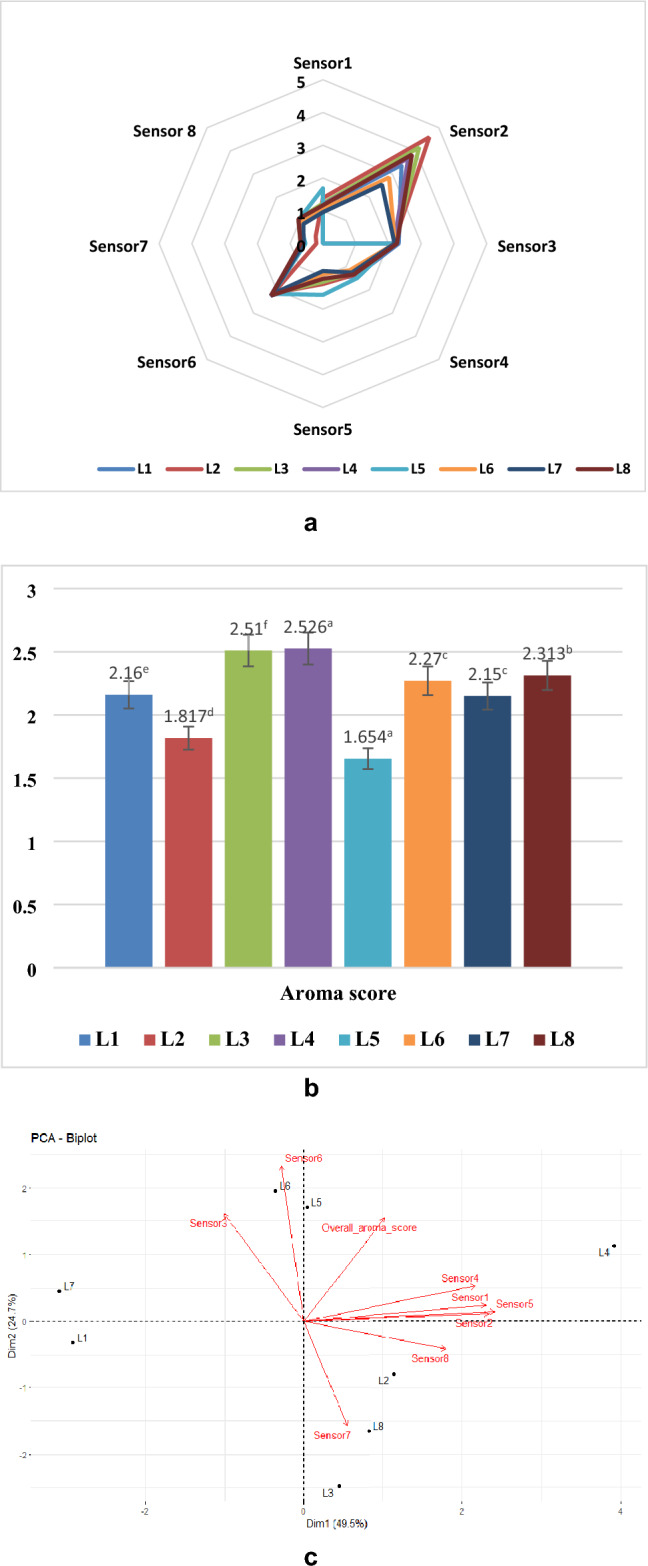
(**a**) E-nose sensor response of *Mushk Budiji* rice grown at different locations. (**b**) Aroma score of *Mushk Budiji* rice grown at different locations. (**c**) PCA plot of *Mushk Budiji* rice grown at different locations using E-nose.

The volatile profile of *Mushk Budiji* rice grown at 8 different locations was characterized using electronic nose (E-nose). This device contains an array of sensors that respond to different types of volatile compounds present in the sample. Radar plot depicted in Fig. 2a shows almost a similar shape for all the samples, indicating that majority of aromatic compounds were present in *Mushk Budiji* rice samples grown at different altitudes. Rice samples of each location showed highest sensor response for sensor 2(TGS-816), detecting the hydrocarbons. The highest sensor response of 1.153 recorded by sensor 4 for L5, indicated the presence of higher percentage of aldehydes (hexanal), alcohols (propylene glycol) and hydrocarbons (n-hexane) in rice samples grown at location 5. These compounds possess relatively lower odour threshold and thus contribute to the rice flavor^20^. Proportionally higher VOC’s were present in rice grown at L5 which was in accordance with the highest sensor response of 2.173 recorded by sensor 6 which is sensitive to detect aldehydes, alcohols and ketones. Based on the different sensitivities and responses of the E-nose sensors, it was presumed that Sensor 6-TGS-2600 positively co-related with hydrocarbons, alcohols & ketones while Sensor 2-TGS-816 and Sensor 3-TGS-823 positively correlated with hydrocarbons (alkanes) and alcohols respectively. Overall the rice samples grown at L4 location showed highest sensor response, which was also validated by GC–MS technique. Aldehydes are mainly produced via lipid oxidation and their decomposition contribute to the overall flavor of aromatic rice because of their relatively lower odour threshold. Alcohols which are amongst the abundant volatiles possess lower odour threshold. They are regarded as the secondary products of unsaturated fatty acid oxidation, formed due to breakdown of aldehydes^20^.

Overall higher E-nose scores were recorded for *Mushk Budiji* grown at lower altitudes as compared to higher altitudes, which indicated that flavor changes occurrence were more at higher altitudes as compared to lower altitudes^31^. Out of all the eight tested samples highest E-nose score was recorded in L4 (2.52) followed by L3 (2.51) (as shown in Fig. 2b), which is in accordance to the flavor profiling results obtained through GC–MS.

### Principal component analysis of sensor response from E-nose

Principal component analysis (PCA) was done to identify the differences in the flavor profile of *Mushk Budiji* grown at eight different locations. Data set obtained from E-nose comprising of sensor response from eight sensors and overall aroma score was analyzed in reduced dimension (Fig. 2c). Principal component 1 (Dimension 1) and 2 (Dimension 2), reflecting the abscissa and ordinate of the biplot in Fig. 2c accounted for 49.5% and 24.7%, of total variability respectively. The sensor response of location 4 was much higher than other locations as reflected by the numerical difference on the abscissa. All the sensors showed positive correlation with each other and sensor 5 (sensitive for hydrocarbons) showed higher variability. Sensor 3, 6 and 7 showed comparatively lesser variability as reflected by the PCA biplot.

### Free fatty acid content

**Figure 3:**
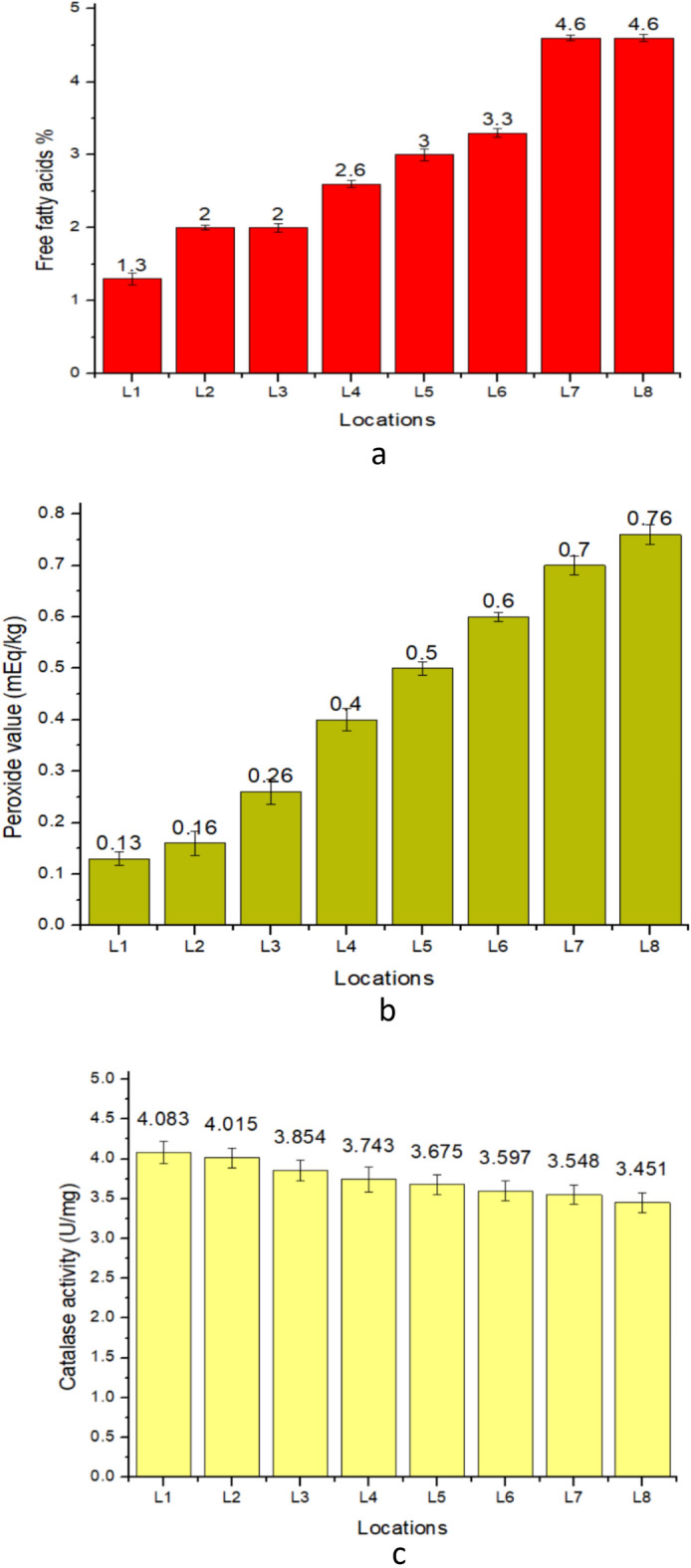
Effect of different locations on a) free fatty acid, b) peroxide value & c) catalase activity of *Mushq Budiji* rice.

Lipases are naturally present in rice and can hydrolyze rice lipids to produce free fatty acids (FFA), which negatively impacts rice quality^33^. FFA content of the *Mushk Budiji* collected from 8 different locations showed significant (p ≤ 0.05) variation. As FFA content showed an increasing trend from L1 (1.3%) to L8 (4.6%) as shown in (Fig. 3a). Lesser amount of FFA content was recorded in lower altitudes samples as compared to higher altitude areas, possibly due to temperature difference. Biao et al.^31^ also reported that high temperature accelerates the free fatty acid generation in rice.

### Peroxide value

Peroxide value (PV) indicates the amount of peroxides formed in fats and oils during oxidation^34^ and is an important indicator of primary lipid oxidation. Although, PV is not a reliable index to judge the rancidity, but fat is generally considered rancid at PV of greater than 10. It has been reported that peroxide formation would increase in presence of high temperature, indicating that oxidation of fats in the product. The peroxide values of *Mushk Budiji* grown at eight different locations also showed significant (p ≤ 0.05) variation. PV increased from 0.13 meq/kg in L1 to 0.76 meq/kg in L8 (Fig. 3b) possibly due to increase in temeperature as higher peroxide fraction occurs at high temperature. This could be attributed to the fact that higher altitude has lesser temperature in comparison to lower altitudes. Biao et al.^36^ also reported that high temperature causes a surge in peroxide value. The range of peroxide value (0.13 meq/kg- 0.76 meq/ kg) recorded in this study was in accordance with the results reported previously by Ozkan et al.^34^ for fats and oils.

### Catalase activity

Catalase is an enzyme capable of decomposing hydrogen peroxide (H_2_O_2_) into water (H_2_O) and oxygen (O_2_), which can reduce the ability of peroxides to promote lipid oxidation^31^. In our study, catalase activity of *Mushk Budiji* rice significantly decreased (p ≤ 0.05) from L1 (4.083U/mg) to L8 (3.451U/mg) (Fig. 3c). This is due to the fact that lower altitude might inhibit the catalase activity present in rice due to the higher temperatures^36^.

## Conclusion

The preamble of the results indicated that aroma profile of *Mushk Budiji* (an indigenous aromatic rice variety) varied with altitude and climatic conditions. In general, higher concentrations of aldehydes, alcohols, and esters, majorly recognized as aroma triggering compounds were recorded in *Mushk Budiji* grown at L8 location (altitude 5216.53 ft; temperature 29 °C), while 2-AP was detected only in *Mushk Budiji* samples grown at L1 & L3. Significant variation in E-nose score was also observed in *Mushk Budiji* samples grown at different altitudes and highest E-nose score (2.52) was recorded for L4. Principle component analysis method used accurately reflected the differences in the flavor profiles of *Mushk Budiji* grown at different altitudes. Therefore, it was presumed that altitude together with low temperature stimulates the accumulation of 2-AP in aromatic rice. Based on the findings of our study, it was concluded that besides 2-AP, several other VOC’s, particularly aldehydes, alcohols and hydrocarbons also contributed to the aromaticity of *Mushk Budiji* rice grown at various altitudes in Highland Himalayan region. Relationship between the flavor metabolic pathways and gene expression w.r.t altitudes was also explored in the study. High altitude was found to promote the over expression of fatty acid degradation and linoleic acid metabolism genes.

## Methods

### Materials

*Mushk Budiji* seeds (an indigenious aromatic rice varietry) were procured from Mountain Research Centre for Field Crops (MRCFC), Khudwani, J&K, India and all the methods used in this work are in compliance with the institutional guidelines. The seeds were sown at 8 different locations of Jammu & Kashmir, India, viz, L1- (Arwah-Budgam; Altitude- 7053.80 ft), L2-(Sagam- Anantnag; Altitude- 6397.63 ft), L3-(Meeliyal- Kupwara; Altitude- 6328.74 ft), L4-(Satura- Pulwama; Altitude- 6299.21 ft), L5-(Chandilura- Baramulla; Altitude- 6167.97 ft), L6-(Khudwani-Anantnag; Altitude- 5314.96 ft), L7-(Kachwamuqam- Baramulla; Altitude- 5226 ft), L8-(Wadura- Sopore; Altitude- 5216.53 ft), in the month of June. The altitude range was chosen in order to identify the suitable location for the production of volatile aromatic compounds in *Mushk Budiji* rice. Transplanting was done using 25 days old seedling using 2–3 plants/hill with row to plant spacing of 15 × 15 cm. The fertilizer doses of nitrogen, phosphorous, potassium (NPK) & ZnSO_4_ recommended as (N:P:K: ZnSO_4_) (70:90:35:15) kg/ha was applied. Soil status of all the locations along with the mean temperature, and mean rainfall from June (2021) to October (2021) is depicted in Table S1. Harvesting was done at the initial physiological maturity stage, when rice grains turned into brown color. Standard agronomic practices were followed in collecting *Mushk Budiji* in all the selected locations. The paddy samples (Fig. S1a) collected from eight different locations were dehusked in THU-34A Satake Testing rice husker (Satake, Japan) and brown rice obtained thereof was polished in a BS08A Satake Single pass friction rice pearler (Satake, Japan) for 1 min. The polishing of samples was done to remove the outer layers (bran and germ) of rice, since *Mushk Budiji* is consumed after polishing. The milled rice samples (Fig. S1b) were packed in plastic containers and stored at 4 ± 1 °C in separate containers until analyzed. Chemicals, complementary DNA (CDNA) synthesis kit, primers and housekeeping genes used in the study were purchased from Sigma-Aldrich, USA.

### Gas chromatography mass spectroscopy analysis

Gas chromatography mass spectroscopy (GC–MS) analysis for identification and quantification of volatile aroma compounds was done as per the method reported by Mahattanatawee et al.^35^. Weighed mass of rice sample (2 g) was placed into head space vial and then 5 ml of type I water was added. The vial was then sealed with a silicon cap and was equilibrated for 30 min at 80 °C with shaking level-3 for further injection into GC–MS.

GC–MS analysis was performed on GC–MS-TQ8040 instrument (GC–MS-TQ Shimadzu, Japan) using Stabilwax capillary column (internal diameter-30 m × 0.25 mm and0.25 µm film thickness). The column temperature was initially held at 50 °C for 1 min followed by 200 °C at 15 ° min^−1^ and 220 °C at 5°Cmin^-1^. The column temperature was held at 220 °C for 5 min. The injector was maintained at 230 °C and 1 µl of sample was injected in splitless mode. Ultra-high purity helium (99.99%) was employed as carrier gas at a constant flow of 2.6 ml min^−1^. The transfer line and electron ionization (EI) source temperature was set at 230 °C, and quadrupole mass analyzer temperature was set at 150 °C. Data acquisition was performed in scan range of 35–500 m/z. The contents of volatiles components were quantified by measuring the peak areas in the total ion chromatogram (TIC).

### Gene expression analysis

In order to analyze the effects of different locations on the gene expression in *Mushk Budiji* rice. Rice panicle/tissue from all the selected locations were collected and stored in RNA later till further analysis. The total RNA was extracted from 100 mg fresh weight of embryonic tissues using Trizol reagent (Invitrogen)^36^. This was followed by treatment of RNase-free DNase I (Sigma-Aldrich, USA). CDNA synthesis was carried out by Thermo Fisher Scientific RevertAid First Strand CDNA Synthesis Kit using oligodT primers, as per the manufacturer’s protocol. The flavor controlling genes and their primer sequence used in the study is given in Table S2. Relative quantification of genes was done by quantitative real-time PCR of normalized CDNA using Roche FastStart Universal SYBR Green Master (Rox) (Roche). Values were calculated using 2^-△△CT^ relative quantification method^37^ for candidate genes and UBQ5 as reference gene. Relative expression studies were done for genes related to fatty acid, linoleic acid and ether lipid metabolism. C_t_ (cycle threshold) curve was obtained from quantitative Real Time-PCR for all the eight selected locations. C_t_ values were used to calculate ΔC_t_ for both test and the positive calibrator (L3), where ΔC_t_ equals [C_t_ (target gene) − C_t_ (reference gene)]. Afterward, ΔΔC_t_ was calculated as: ΔΔC_t_ = ΔC_t_ gene (location) − ΔC_t_ (location3). The ΔΔC_t_, so obtained, was translated to yield relative fold change (2 − ΔΔC_t_) in expression of target genes.

### E-nose analysis for aroma detection

**Figure 4:**
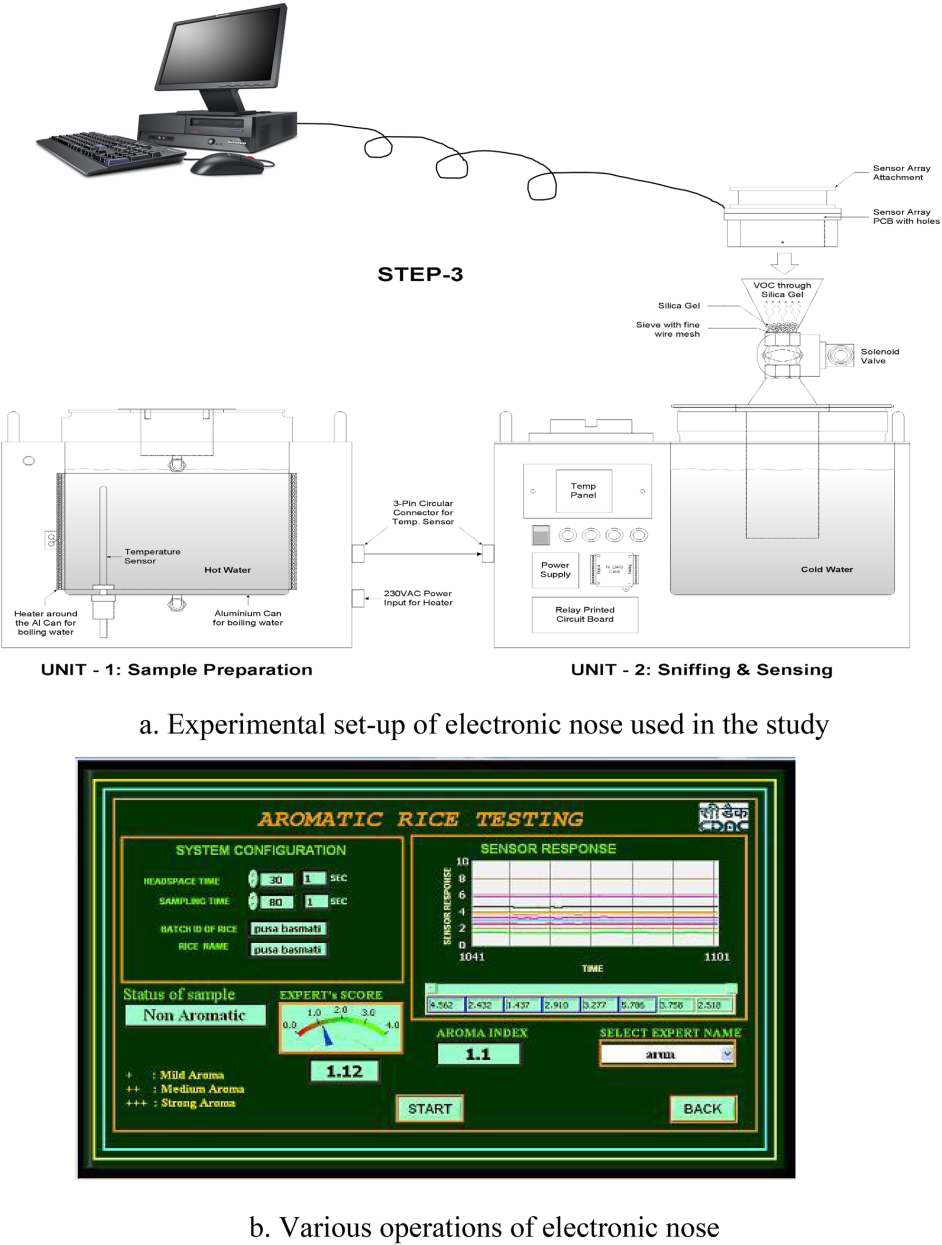
(**a**) Experimental set-up of electronic nose used in the study. (**b**) Various operations of electronic nose.

Electronic nose detector with metal-oxide semiconductor (MOS)-based gas analyzer array designed by Centre for Development of Advanced Computing (C-DAC, Kolkatta, India) was used in this study. This device contained an array of 8 different non specific commercial tin oxide semiconductor sensors from Figaro, Japan namely- Sensor 1-TGS-825 (sensitive for sulphur containing compounds), Sensor 2-TGS-816 (sensitive for hydrocarbarbons- alkanes), Sensor 3-TGS-823 (organic solvents such as alcohols), Sensor 4-TGS-832 (hydrocarbons- halocarbons), Sensor 5-TGS-830 (sensitive for hydrocarbons), Sensor 6-TGS-2600 (sensitive for hydrocarbons, alcohols, ketones), Sensor 7-TGS- 2620 (sensitive for hydrocarbons, organic solvents such as alcohols), and Sensor 8-TGS-821 (sensitive for hydrocarbons) to discriminate between the odour patterns of different aromatic compounds. The experimental set-up of electronic nose for aromatic rice system consists of: (1) a sensor array; (2) a micro-pump and solenoid valves with programmable sequence control; (3) PC-based data acquisition; and (4) olfaction software. Specially designed sample holders made of aluminum were used for experimental runs. An aluminum sample holder was fixed to the instrument by simple lock fitting. The entire sniffing cycle consisted of an automated sequence of internal operations, viz., (1) headspace generation, (2) sampling, and (3) purging (Fig. 4a).

Rice samples (15 g) were placed in an aluminum sample holder to which 60 ml of distilled water was added. Samples were cooked in the E-nose setup at a temperature of 100 °C for 20 min. The cooked rice samples were placed for cooling in the sniffing chamber for 10 min. Prior to sampling the adequate accumulation of volatile compounds was ensured in head space of the sniffing chamber. The sensor array was then exposed to a constant flow (150 ml/min) of volatiles through pipelines inside the apparatus. During the purging operation, sensor heads were cleared with a blow of fresh air to bring the sensors back to the baseline values. The stepwise sequence of operations followed during the experimental sniffing cycle of E-nose are depicted in Table S3 and Fig. 4b.

### Free fatty acid content

Free fatty acid (FFA) content of rice samples was determined using standard titrimetric method^38^. Ground rice flour (10 g) was placed in whattmann filter paper and oil extraction was done for 16 h using petroleum ether (200 ml) at room temp (24 ± 5 °C). The petroleum ether was evaporated by heating under fume. After evaporation of solvent (petroleum ether), approximately 50 ml of benzene alcohol phenolphthalein solution was added into the oil sample. The sample was titrated with 0.0178N potassium hydroxide (KOH) until faint pink color persisted for 1 min. The FFA content was calculated as:

1 $${\text{FFA}}\left(\mathrm{\%}\right)=\frac{10\times \left(mL\, KOH \,used\right)\times \left(mL\, KOH\, blank\right)}{100\, (g\, water\, in\, 100g\, sample)}\times 100$$

### Peroxide value

Peroxide value of rice samples was analyzed by method reported by Gichau et al.^39^. Soxhlet method was used to extract the lipids from rice samples. Two grams of samples were weighed into 250 ml stoppered conical flask. Thirty milliliter of acetic acid and chloroform solvent mixture (30:20) was added to the sample and swirled to dissolve. Afterwards, 0.5 ml saturated potassium iodide solution was added and left to stand for 1 min in the dark with occasional shaking, followed by addition of 30 ml of distilled water. The mixture was titrated with 0.01 N sodium thiosulphate solution, with vigorous shaking until yellow color disappeared. 0.5 ml starch solution was added as an indicator and titration was continued until the blue color disappeared. Peroxide value was calculated using the following formulae:

2 $$\mathrm{PV\, }({\text{meq}}/{\text{kg}}=\frac{Titre\, value\times N\times 100}{W}$$

where, W is the weight of the sample, Titre value = ml of sodium thiosulphate solution used, N = Normality of sodium thiosulphate solution.

### Catalase activity

Catalase activity was determined using the method reported by Palmiano et al.^40^. Grains were homogenised for 3 min with 10 ml of HCl buffer (pH 7). The homogenate was centrifuged at 10,000 rpm for 30 min at 4 °C. 5 ml of distilled water, 1 ml of Hydrogen peroxide (H_2_O_2_) substrate, and 1 ml of enzyme extract was taken in a conical flask. The mixture was incubated in an incubator at 28 ± 1 °C for 15 min. Finally, the enzyme activity was stopped by adding 5 ml of 10% sulphuric acid (H_2_SO_4_) to the reaction mixture. The residual H_2_O_2_ content of the mixture was estimated through titration against potassium permanganate (KMnO_4_) solution. A control set titration was also made without the addition of enzyme to the reaction mixture (6 ml distill water and 1 ml H_2_O_2_ substrate). The control set was also incubated at 28 ± 1 °C for 15 min and the reaction was stopped by the addition of 5 ml of 10% H_2_SO_4_ before titration against potassium permanganate (KMnO_4_) solution. The difference between the titration reading of the control set (without enzyme) and reaction mixture (with enzyme) provided the amount of H_2_O_2_ decomposed by the enzyme action.

### Statistical models

All the experiments were conducted in triplicates and results were expressed as mean ± standard deviation. The mean differences were analyzed by one way analysis of variance (ANOVA). Statistical significance of means was accessed using Duncan’s Multiple Range test (DMRT) test at p ≤ 0.05 level of significance using SPSS software. In addition, the output data from the E-nose was analyzed using the instrument software of the electronic nose (Lab VIEW). Principle component analysis was performed on the output data of E-nose by XLSTAT trial version (2019).

### Supplementary Information

Supplementary Information.

## Data Availability

Raw data shall be made available on request to corresponding author.

## References

[1] Sakthivel K (2009). Genetic and molecular basis of fragrance in rice. Biotechnol. Adv..

[2] Prodhan ZH (2017). Agronomic, transcriptomic and metabolomic expression analysis of aroma gene (badh2) under different temperature regimes in rice. Biotechnol. Adv..

[3] Wakte K (2017). Thirty‐three years of 2‐acetyl‐1‐pyrroline, a principal basmati aroma compound in scented rice (Oryza sativa L.): A status review. J. Sci. Food Agric..

[4] Varatharajan, N. *et al*. Rice aroma: Biochemical, genetics and molecular aspects and its extraction and quantification methods. *Integrative Advances in Rice Research* (2021).

[5] Ashokkumar S (2020). Creation of novel alleles of fragrance gene OsBADH2 in rice through CRISPR/Cas9 mediated gene editing. PLoS ONE.

[6] Ramtekey V (2021). Extraction, characterization, quantification, and application of volatile aromatic compounds from Asian rice cultivars. Rev. Anal. Chem..

[7] Routray W, Rayaguru K (2018). 2-Acetyl-1-pyrroline: A key aroma component of aromatic rice and other food products. Food Rev. Intl..

[8] Hinge VR, Patil HB, Nadaf AB (2016). Aroma volatile analyses and 2AP characterization at various developmental stages in Basmati and Non-Basmati scented rice (Oryza sativa L.) cultivars. Rice.

[9] Caldana C (2007). A quantitative RT-PCR platform for high-throughput expression profiling of 2500 rice transcription factors. Plant Methods.

[10] Hinge V, Patil H, Nadaf A (2016). Comparative characterization of aroma volatiles and related gene expression analysis at vegetative and mature stages in basmati and non-basmati rice (Oryza sativa L.) cultivars. Appl. Biochem. Biotechnol..

[11] Jona J (2022). Electronic noses and their applications for sensory and analytical measurements in the waste management plants: A review. Sensors.

[12] Lee-Rangel HA (2022). Application of an electronic nose and HS-SPME/GC-MS to determine volatile organic compounds in fresh mexican cheese. Foods.

[13] Tan J, Jie Xu (2020). Applications of electronic nose (e-nose) and electronic tongue (e-tongue) in food quality-related properties determination: A review. Artif. Intell. Agric..

[14] Jana A (2015). Fragrance measurement of scented rice using electronic nose. Int. J. Smart Sens. Intell. Syst..

[15] Zheng X-Z (2009). Rapid identification of rice samples using an electronic nose. J. Bionic Eng..

[16] Zhao Q, Xue Y, Shen Q (2020). Changes in the major aroma-active compounds and taste components of Jasmine rice during storage. Food Res. Int..

[17] Sharma, A. *et al*. *Aromatic Rice of India: It’s Types and Breeding Strategies* (IntechOpen, 2021).

[18] Khan GH (2018). Marker-assisted introgression of three dominant blast resistance genes into an aromatic rice cultivar Mushk Budji. Sci. Rep..

[19] Sansenya S, Wechakorn K (2021). Effect of rainfall and altitude on the 2-acetyl-1-pyrroline and volatile compounds profile of black glutinous rice (Thai upland rice). J. Sci. Food Agric..

[20] Hu X (2020). Volatile compounds, affecting factors and evaluation methods for rice aroma: A review. Trends Food Sci. Technol..

[21] Zeng M (2012). Determination of flavor components of rice bran by GC-MS and chemometrics. Anal. Methods.

[22] Jie Y (2021). Identification of key volatiles differentiating aromatic rice cultivars using an untargeted metabolomics approach. Metabolites.

[23] Verma DK, Srivastav PP (2020). A paradigm of volatile aroma compounds in rice and their product with extraction and identification methods: A comprehensive review. Food Res. Int..

[24] Chen Z (2020). Metabolomic analysis reveals metabolites and pathways involved in grain quality traits of high-quality rice cultivars under a dry cultivation system. Food Chem..

[25] Zeng Y (2019). Changes in the rice grain quality of different high-quality rice varieties released in southern China from 2007 to 2017. J. Cereal Sci..

[26] Yang DS (2008). Comparison of odor-active compounds from six distinctly different rice flavor types. J. Agric. Food Chem..

[27] Yoshihashi T, Nguyen TTH, Kabaki N (2004). Area dependency of 2-acetyl-1-pyrroline content in an aromatic rice variety, Khao Dawk Mali 105. Jpn. Agric. Res. Q. JARQ.

[28] Medda S, Fadda A, Mulas M (2022). Influence of climate change on metabolism and biological characteristics in perennial woody fruit crops in the Mediterranean environment. Horticulturae.

[29] Bao G (2018). Molecular basis for increased 2-acetyl-1-pyrroline contents under alternate wetting and drying (AWD) conditions in fragrant rice. Plant Physiol. Biochem..

[30] Prodhan ZH, Qingyao SHU (2020). Rice aroma: A natural gift comes with price and the way forward. Rice Sci..

[31] Biao Y (2019). Influence of gene regulation on rice quality: Impact of storage temperature and humidity on flavor profile. Food Chem..

[32] Lee KJ (2016). Genome-wide expression analysis of a rice mutant line under salt stress. Genet. Mol. Res..

[33] Zhou Z (2002). Ageing of stored rice: Changes in chemical and physical attributes. J. Cereal Sci..

[34] Ozkan G, Simsek B, Kuleasan H (2007). Antioxidant activities of Satureja cilicica essential oil in butter and in vitro. J. Food Eng..

[35] Mahattanatawee K, Rouseff RL (2014). Comparison of aroma active and sulfur volatiles in three fragrant rice cultivars using GC–Olfactometry and GC–PFPD. Food Chem..

[36] Chomczynski P, Sacchi N (1987). Single-step method of RNA isolation by acid guanidinium thiocyanate-phenol-chloroform extraction. Anal. Biochem..

[37] Livak KJ, Schmittgen TD (2001). Analysis of relative gene expression data using real-time quantitative PCR and the 2− ΔΔCT method. Methods.

[38] American Association of Cereal Chemists. *Approved Methods Committee. Approved Methods of the American Association of Cereal Chemists* (AACC, 2000).

[39] Gichau AW (2019). Use of peroxide value and moisture content as a measure of quality for amaranth-sorghum grains based complementary food. Nutr. Food Technol..

[40] Palmiano EP, Juliano BO (1973). Changes in the activity of some hydrolases, peroxidase, and catalase in the rice seed during germination. Plant Physiol..

