# Supplementary material for: Flavor profiling and gene expression studies of indigenous aromatic rice variety (Mushk Budiji) grown at different altitudes of Highland Himalayan regions
Source: Sci Rep. 2024 Jan 10;14:1010. doi: 10.1038/s41598-024-51467-z (PMC10781667; doi:10.1038/s41598-024-51467-z)
Supplement: Supplementary file 1 — Supplementary Information. [file 41598_2024_51467_MOESM1_ESM.docx]

**Table S1: Temperature, Rainfall and Soil status of all the 8 tested locations**

| **Locations** | **Altitude (ft)** | **Temperature (°C)** | | **Rainfall (mm)** | **Avaibale nitrogen (N)kg/ha** | **pH** |
| --- | --- | --- | --- | --- | --- | --- |
|  |  | **Max** | **Min** |  |  |  |
| **Arwah (L1)** | 7053.80 | 17.44±2.70 | 8.57±1.75 | 74.29±6.68 | 420 | 6.8 |
| **Sagam (L2)** | 6397.63 | 26.1±2.96 | 13.3±1.73 | 69.21±6.42 | 380 | 6.7 |
| **Kupwara (L3)** | 6328.74 | 26.92±3.07 | 13.62±1.98 | 67.94±5.05 | 215 | 6.6 |
| **Satura (L4)** | 6299.21 | 27.09±3.21 | 13.71±2.01 | 58.42±4.55 | 421 | 6.2 |
| **Chandilura (L5)** | 6167.97 | 28±2.44 | 13.17±1.92 | 43.81±5.3 | 380.5 | 7.2 |
| **Khudwani (L6)** | 5314.96 | 28.51±3.20 | 16.02±2.04 | 43.63±5.2 | 215 | 7.2 |
| **Kachwamuqam (L7)** | 5226 | 28.93±3.30 | 8.57±1.65 | 41.14±4.8 | 290 | 6.3 |
| **Wadura (L8)** | 5216.53 | 29±3.34 | 8.57±1.62 | 41.10±4.7 | 285 | 6.8 |

*Temp and rainfall data is the average readings from June 2021- October 2021

**Table S2: Primer sequence of 10 different genes used in the qRT-PCR analysis of *Mushk Budiji* rice**

| **Genes** | **Sequence** |
| --- | --- |
| Gene 1: OS11G0605500 | F:ATTGGTGTGCGACCTTTACG |
|  | R:TTGGGGCAACATAGTCCACA |
| Gene 2: OS05G0132100 | F:AACAGGGTGATACTGCCAGC |
|  | R:GACCAAAGTGACCCCCAGAG |
| Gene 3: OS08G0508800 | F:TGATGAAGCCCTTCTCCGAC |
|  | R:TTTGCAAGTAGTTTTGGAGCG |
| Gene 4: OS03G0738600 | F:GTCGACCCCAACAATGGGAA |
|  | R:CGAACTTGGACTCTCCCGTC |
| Gene 5: OS08G0509100 | F:GAAGCTCAAGAATCGGTGCG |
|  | R:TCACACACATGATGACATGAACG |
| Gene 6: OS06G0604400 | F:GCACGACATCCACTCCAAGA |
|  | R:CGATGGACCGGAACAACTGA |
| Gene 7: OS02G0676000 | F:CTGGCACCGGCTCTTCTATC |
|  | R:TTTGCACGATCCCATTTGGC |
| Gene 8: OS03G0826600 | F:TATGACCATGTGCCTACGCC |
|  | R:TCATGGATCACAGTGCGCTT |
| Gene 9: OS09G0543100 | F:ATCGGTGGCCTGGATCTTTG |
|  | R:GCAGCTGGACCATCAATCCT |
| Gene 10: badh2 | F:TGTGCTAAACATAGTGACTGGA |
|  | R:CTTAACCATAGGAGCAGCT |
| Gene 11: UBQ5 (REF) | F: ACCACTTCGACCGCCACTACT |
|  | R: ACGCCTAAGCCTGCTGGTT |

**Table S3. Sequence of operations in an experimental sniffing cycle of E-nose**

| **Operation** | **Purpose** | **Duration** |
| --- | --- | --- |
| Cooking | To cook rice sample at 100^o^ C | 20 min |
| Cooling | To cool down the rice sample at room temperature | 10 min |
| Headspace generation | To accumulate adequate volatile compounds before sampling | 30 sec |
| Sampling | Exposure of the sensor array to the volatiles of aromatic rice. | 80 sec |
| Purging | Cleaning the sensor surface with blow of fresh air so that the sensor output returns to the baseline value. | 300 sec |


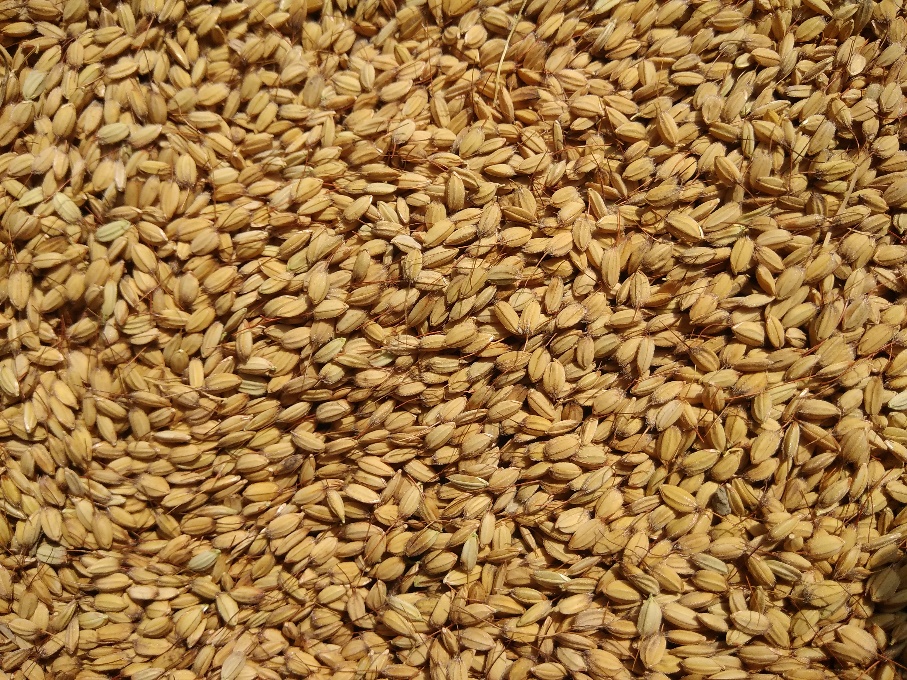


a)


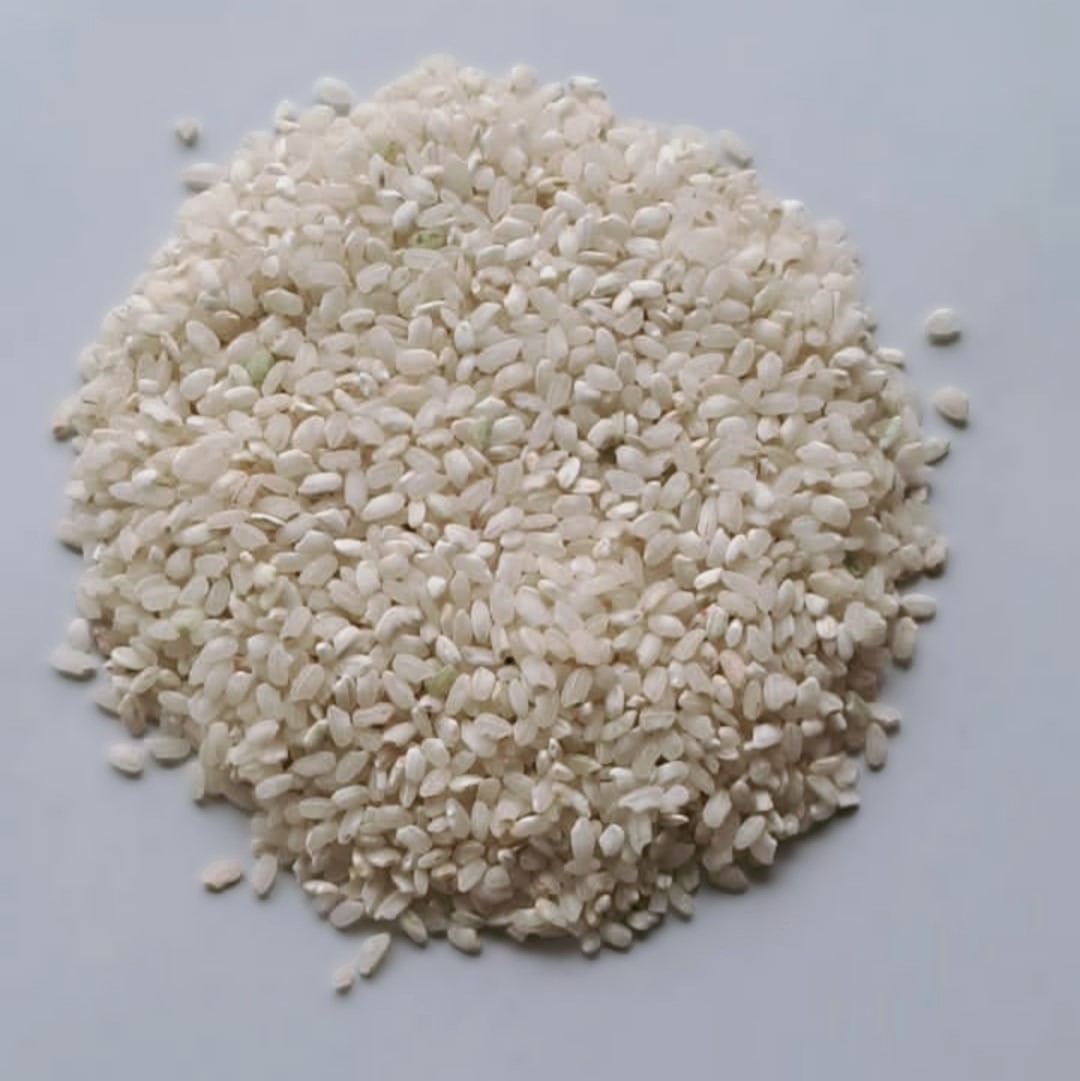


b)

Figure S1: Pictorial representation of a) undehusked *Mushk budiji* b) milled *Mushk budiji*
